# Genome-wide association study identifies loci for arterial stiffness index in 127,121 UK Biobank participants

**DOI:** 10.1038/s41598-019-45703-0

**Published:** 2019-06-24

**Authors:** Kenneth Fung, Julia Ramírez, Helen R. Warren, Nay Aung, Aaron M. Lee, Evan Tzanis, Steffen E. Petersen, Patricia B. Munroe

**Affiliations:** 1 Advanced Cardiovascular Imaging, William Harvey Research Institute, Queen Mary University of London, Charterhouse Square, London, UK; 2 Clinical Pharmacology, William Harvey Research Institute, Queen Mary University of London, Charterhouse Square, London, UK; 3 NIHR Barts Biomedical Research Centre, Queen Mary University of London, Charterhouse Square, London, UK

**Keywords:** Genome-wide association studies, Cardiovascular genetics

## Abstract

Arterial stiffness index (ASI) is a non-invasive measure of arterial stiffness using infra-red finger sensors (photoplethysmography). It is a well-suited measure for large populations as it is relatively inexpensive to perform, and data can be acquired within seconds. These features raise interest in using ASI as a tool to estimate cardiovascular disease risk as prior work demonstrates increased arterial stiffness is associated with elevated systolic blood pressure, and ASI is predictive of cardiovascular disease and mortality. We conducted genome-wide association studies (GWASs) for ASI in 127,121 UK Biobank participants of European-ancestry. Our primary analyses identified variants at four loci reaching genome-wide significance (*P* < 5 × 10^−8^): *TEX41* (rs1006923; *P* = 5.3 × 10^−12^), *FOXO1* (rs7331212; *P* = 2.2 × 10^−11^), *C1orf21* (rs1930290, *P* = 1.1 × 10^−8^) and *MRVI1* (rs10840457, *P* = 3.4 × 10^−8^). Gene-based testing revealed three significant genes, the most significant gene was *COL4A2* (*P* = 1.41 × 10^−8^) encoding type IV collagen. Other candidate genes at associated loci were also involved in smooth muscle tone regulation. Our findings provide new information for understanding the development of arterial stiffness.

## Introduction

Arterial stiffness measures have been reported as independent markers of vascular ageing^1,2^, hypertension^3,4^, cardiovascular disease (CVD)^5,6^ and mortality^6,7^. Carotid-femoral pulse wave velocity (PWV) is the reference standard method for measuring arterial stiffness. However, an alternative and more convenient non-invasive method is to record the digital blood volume waveforms using infra-red finger sensors (photoplethysmography)^8^, where measurements can be recorded in a seated position rather than supine position required for carotid-femoral PWV recordings. This automatic technique is able to detect the waveform formed by the digital volume pulse, which is created by two components. First, pressure is transmitted from the left ventricle to the finger (direct component) whilst the second component is due to the transmission of pressure from the heart to the lower body (reflected component) via the aorta. The digital volume pulse can therefore be visualised as a dicrotic waveform, and the interval between the peaks of the direct and reflected components can be recorded to derive the arterial stiffness index (ASI), when divided into the individual’s height. Higher ASI values reflect arterial walls with greater stiffness, due to the earlier arrival of wave reflection. ASI has been shown to have close agreement with other techniques measuring arterial stiffness, including PWV (r = 0.58, *P* < 0.01) and augmentation index (r = 0.80, *P* < 0.01)^9^.

Previous studies have evaluated the utility of ASI as a potential clinical marker of CVD, having shown good sensitivity (87%) and specificity (87%) when differentiating between older men with coronary artery disease (CAD) and younger men without CAD^9^. In a study of asymptomatic middle-aged patients, the mean ASI value was significantly higher (*P* = 0.002) in individuals with at least one significant (>50%) coronary stenotic plaque than those without a >50% stenotic lesion^10^. More recently, ASI has been shown to be an independent predictor of CVD, myocardial infarction and mortality in the UK Biobank cohort^11^. During a median follow-up period of 2.8 years, the risk of CVD and myocardial infarction for individuals with higher ASI were 27% and 38% higher respectively. For all-cause mortality, each standard deviation change in ASI has a hazard ratio of 1.08 (95% confidence interval [CI], 1.05–1.12) in a multi-variant adjusted model^11^.

Our current knowledge of the biological factors and pathways contributing to arterial stiffness is limited. Heritability studies of arterial stiffness using PWV measurements have suggested moderate genetic contribution with estimates up to 0.53 in twin studies^12,13^ and ranging between 0.26 and 0.40 in population studies^14,15^. The identification of single nucleotide variants (SNVs) may improve the current understanding of the mechanisms controlling arterial stiffness and may be a useful addition to the development of disease risk models. Previous genome-wide association studies (GWASs) for arterial stiffness were mostly performed using PWV as the phenotype. One of the first arterial stiffness GWASs was on 644 individuals from the Framingham Heart Study, using carotid-brachial PWV as the phenotype, and no variants reached genome-wide significance^16^. Subsequent genetic studies have reported inconsistent findings but none have studied ASI as the phenotypic measure^17^. The wealth of data available within the UK Biobank study offers the opportunity to produce more robust findings in a large population cohort. We therefore performed a GWAS to identify SNVs associated with ASI and explored the biological mechanisms underlying arterial stiffness.

## Results

### Four loci identified for arterial stiffness index

In total, 143,590 UK Biobank participants met the inclusion criteria to derive ASI at the baseline visit (Supplementary Fig. S1). After genetic quality control (QC) exclusions, three GWASs were performed on 127,121 individuals of European ancestry (age 56 ± 8.1, 48.1% males) with a mean ASI of 9.0 m/s. Our primary GWAS was performed on rank based inverse normal transformed (INT) residuals and included mean arterial pressure (MAP) as a covariate. We also performed analyses excluding MAP and on untransformed ASI. The baseline visit characteristics of the individuals included in the GWAS are summarised in Supplementary Table S1. Following the GWAS, we reviewed the results and quantile-quantile (QQ) plots. There was minimal genomic inflation in test statistics (lambda = 1.097 for all models) under polygenic inheritance (Supplementary Fig. S2). The genome-wide SNV heritability of ASI in our cohort was estimated at 6.1% (standard error 0.4%).

**Figure 1:**
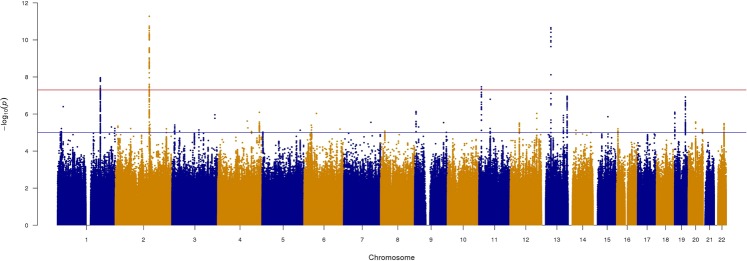
Manhattan plot for arterial stiffness index (ASI) in UK Biobank. The red line indicates the *P*-value threshold for genome-wide significance (5 × 10^−8^) while the blue line indicates *P*-value threshold for suggestive significance (1 × 10^−5^).

**Figure 2:**
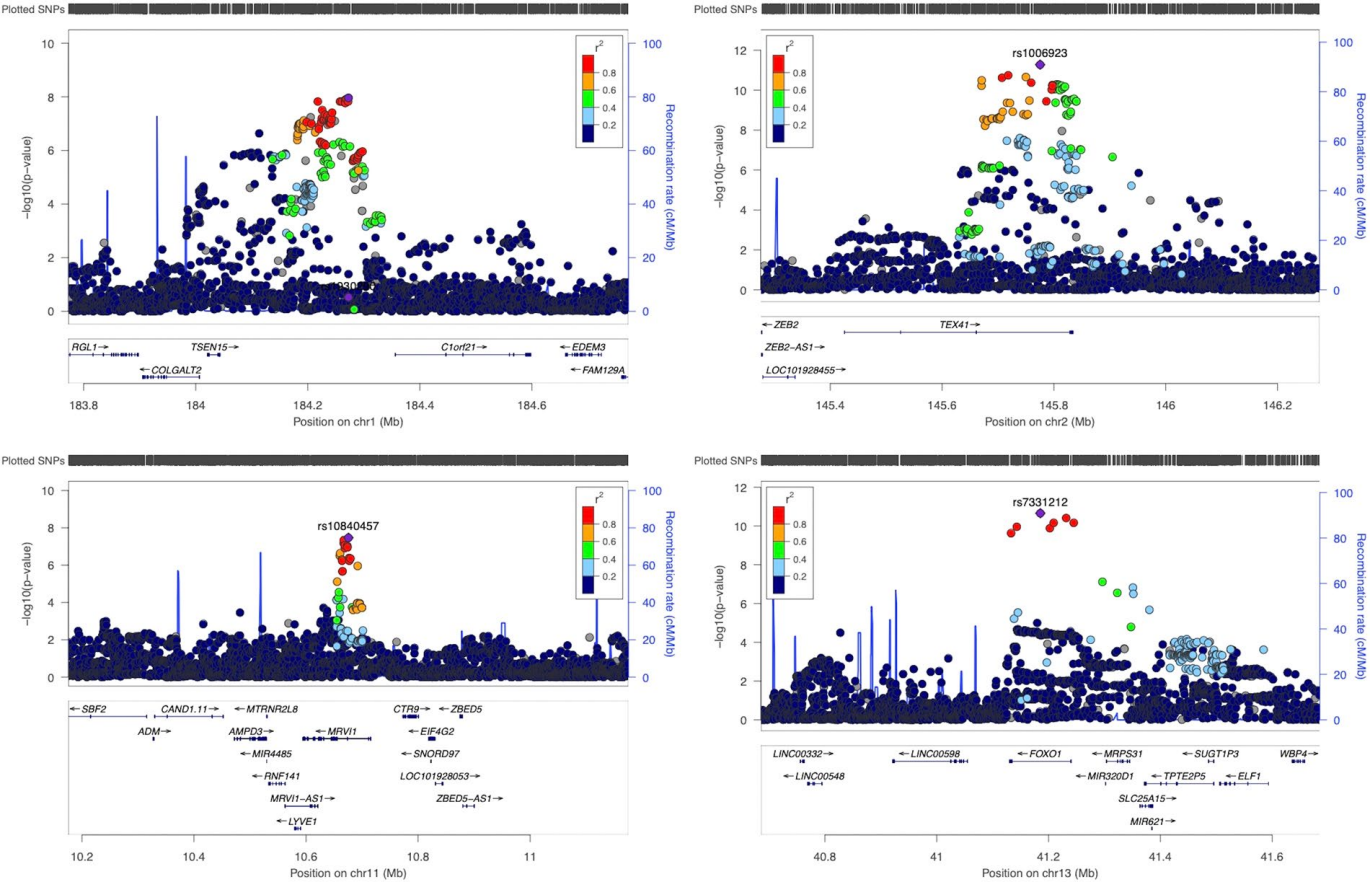
LocusZoom plots for arterial stiffness index loci (*P* < 5 × 10^−8^).

We identified four genome-wide significant loci (*TEX41*, *FOXO1*, *C1orf21 and MRVI1)* for ASI in our primary analysis (Table 1 and Fig. 1). The regional association plots are indicated in Fig. 2. Three of the loci (*TEX41*, *FOXO1* and *C1orf21*) were genome-wide significant in the secondary GWAS that excluded the MAP adjustment (Supplementary Table S2a and Supplementary Fig. S3) and in the GWAS of untransformed ASI (Supplementary Table S2b). The magnitude of effect sizes and associations of the significant loci were similar across the primary and secondary models. We observed no independent signals at any of the ASI loci.

**Table 1:** Summary of genome-wide significant loci associated with arterial stiffness index.

Locus	SNV	CHR	BP	EA	EAF	P	β	SE
C1orf21	rs1930290	1	184272584	T	0.553	1.1E-08	−0.023	0.004
TEX41	rs1006923	2	145775399	T	0.677	5.3E-12	0.029	0.004
MRVI1	rs10840457	11	10675738	A	0.314	3.4E-08	−0.024	0.004
FOXO1	rs7331212	13	41185309	G	0.737	2.2E-11	0.030	0.005

Locus indicates the name of the gene in closest proximity to the most significant SNV.

SNV = single-nucleotide variant; CHR = chromosome; BP = base pair position (Build 37); EA = effect allele; EAF = effect allele frequency; P = *P*-value (standard infinitesimal mixed model); β = effect-size estimates on an inverse-normal transformed scale. SE = standard error.

The most significant associated variants in our primary GWAS were: rs1006923, an intronic variant at *TEX41* (β = 0.0293, *P* = 5.3 × 10^−12^), rs7331212 an intronic variant at *FOXO1* (β = 0.0301, *P* = 2.2 × 10^−11^) and rs1930290 (β = −0.0230, *P* = 1.1 × 10^−8^), located in the gene region of the open reading frame *C1orf21*. The intronic variant rs10840457 within the *MRVI1* gene region on chromosome 11 (β = −0.0236, *P* = 3.4 × 10^−8^) was only genome-wide significant in our primary analysis, however there was some support in the secondary analyses (*P* = 3.3 × 10^−7^) and untransformed ASI (*P* = 5.4 × 10^−8^).

Forty-three variants at non-overlapping loci had suggestive genome-wide significance (*P* < 1 × 10^−5^) with ASI in our primary analysis (Supplementary Table S3). A few variants are located close to potential candidate genes of interest: rs9521719 in the *COL4A2* gene (β = 0.0215, *P* = 1.1 × 10^−8^), rs8107744 in the *RSPH6A* gene (β = −0.0276, *P* = 1.2 × 10^−7^). Other variants of note were rs371147897 found in the *OR4A47* gene region (*P* = 1.6 × 10^−7^), rs9501489 located in *DDR1* (*P* = 4.0 × 10^−6^) and rs149320025 located in the *FUCA1* gene region on chromosome 1 (*P* = 4.0 × 10^−7^).

### Functional annotation of four ASI loci

In our primary analysis, 219 candidate variants were identified by SNP2GENE function in the Functional Mapping and Annotation of Genome-wide Association Studies (FUMA) platform^18^. The majority of SNVs and their proxies (r^2^ > 0.8) were located in introns (63%), 31% were located in intergenic regions, there was one acceptor splice variant at *MRVI1*, rs11042902 and the remainder were located in exons and 3′-untranslated regions (3′-UTRs). Of the 219 variants, 151 variants mapped to the four genome-wide significant loci.

Gene-based analysis, as computed by multi-marker analysis of genomic annotation (MAGMA)^19^, which mapped the output SNVs from BOLT-LMM to 18,666 protein coding genes, identified four genes that reached the gene-wide significance threshold (*P* = 2.67 × 10^−6^). *COL4A2* was the most significant gene for ASI (*P* = 1.41 × 10^−8^) and *MRVI1* (*P* = 3.08 × 10^−7^), *FOXO1* (*P* = 7.54 × 10^−7^) and *FOXO3* (*P* = 2.34 × 10^−6^) were also significant (Supplementary Fig. S4). Two further genes, *TCF20* located 2.9 Mb downstream to rs1006923 (*P* = 5.37 × 10^−6^) and *FBXO46* located on chromosome 19 (*P* = 6.72 × 10^−6^) indicated suggestive significance. The *FBXO46* gene is located less than 67 kb away from rs8107744 at the *RSPH6A* locus on chromosome 19 that was significant from single variant analyses using untransformed ASI values (*P* = 1.4 × 10^−8^, Supplementary Table S2b).

To identify further candidate genes at each locus we reviewed results from expression quantitative trait loci (eQTL) analyses across 53 tissue types from Genotype-Tissue Expression (GTEx) database from FUMA^18^. Significant eQTLs were observed for *SLC25A15* at the *FOXO1* locus across several tissues including oesophagus, transformed fibroblasts and sun exposed skin in the lower leg (lead SNV rs12865518, *P* = 2.04 × 10^−8^), *ZEB2* at the *TEX41* locus in the aorta (lead SNV rs2252383, *P* = 3.85 × 10^−5^) and *C1orf21* and *APOBEC4* at the *C1orf21* locus in nerve and brain tissue (Supplementary Table S4).

We also checked if the genetic variants identified for ASI were associated with other traits using PhenoScanner^20^. Genome-wide associations were observed for variants at three of the four ASI loci. Variants at *TEX41* (rs1006923) and *MRVI1* (rs10840457) were associated with blood pressure traits and ASI from analyses in UK Biobank^21^. Significant associations were also observed with pulse wave peak to peak time (PPT) for rs1006923 (*TEX41*) and rs7331212 at the *FOXO1* locus by the Neale lab^21^ that has publicly released GWAS results for over 4,200 phenotypes found within UK Biobank. The variant at the *TEX41* locus was additionally associated with CAD^22^ (Supplementary Table S5).

## Discussion

Our main finding was the identification of four genome-wide significant loci for ASI in a large European-ancestry based population cohort, despite the relatively low (0.06) heritability observed for ASI. We further followed-up with several candidate genes using bioinformatics analyses at each of the identified loci.

Previous genetic association studies for arterial stiffness, as summarised by Logan *et al*.^17^, have mainly used PWV as the phenotypic measure of stiffness and the reported findings were limited. A meta-analysis including GWAS results of 20,634 individuals from 9 discovery cohorts and of 5,306 individuals from two replication cohorts, all from European ancestry, identified one locus on chromosome 14 in the *3*′*-BCL11B* gene desert. This gene desert was shown to be associated with the carotid-femoral PWV (rs7152623, discovery *P* = 2.8 × 10^−10^, replication *P* = 1.4 × 10^−6^)^23^. However, variants at this locus were not statistically significantly associated with ASI (*P* = 0.08) in our dataset.

More recently, a GWAS for brachial-ankle PWV (baPWV), involving 402 Korean patients (mean age 59 years, 59% male) with diagnosed CVD has been reported by Park *et al*.^24^. Two SNVs were found to be associated with baPWV – rs7271920 (*P* = 7.15 × 10^−9^) and rs10125157 (*P* = 8.25 × 10^−7^). Neither variant was significant (rs7271920, *P = *3.51 × 10^−1^; rs10125157, *P* = 3.10 × 10^−1^) in the replication cohort in their study that included 1,206 individuals. We also observed non-significant results for both variants (rs7271920, *P* = 0.17; rs10125157, *P* = 0.81) in our study. It is not too surprising to observe a lack of significant findings for the two variants reported by Park *et al*.^24^ in the UK Biobank cohort. A contributing factor may be the difference in the size of the populations, and the different ethnicities, and that the reported variants by Park *et al*.^24^ may be false positive findings as there was no replication in their study.

The lack of replication of results on PWV in UK Biobank and across PWV GWASs may also be due to the lack of methodological standardisation to derive PWV, as it can be measured at different sites such as carotid-femoral or brachial-ankle. Our findings suggest that ASI and PWV may have different aetiologies and thus may provide independent data on the underlying biological mechanisms and potential cardiovascular risk factors. A previous expert consensus statement on arterial stiffness described PWV as a measure of regional stiffness, while ASI is seen as a surrogate marker of stiffness through wave reflection assessments^25^. In other words, whilst PWV is determined mainly by the speed at which waveform travels, for ASI, other factors such as the reflective point would also impact on its measurements leading to potential differences in their aetiologies.

Our most significant variant for ASI, rs1006923 at the *TEX41* locus has previously been reported to be significantly associated with CAD in a mixed population GWAS that included UK Biobank participants^22^. It is located 129 kb upstream and in low linkage disequilibrium (LD; r^2^ = 0.19) to rs1438896 a variant reported by Warren *et al*.^26^ with blood pressure traits. Another variant at the *TEX41* locus rs183032 is 50 kb downstream to our lead variant with a moderate LD (r^2^ = 0.38) is associated with aortic stenosis in an Icelandic cohort^27^. Helgadottir (2018)^27^ suggested the tumour growth factor-β (TGF-β) through *ZEB2*^27^ as a candidate gene. ZEB2 has a role as a DNA-binding transcriptional repressor that interacts with the main signal transducers for TGF-β receptors (SMADs). Considering that TGF-β is involved in the regulation of vascular smooth muscle differentiation, as well as in the collagen up-regulation in the vascular wall, changes in the expression of TGF-β have the potential to alter arterial stiffness. We note additional support for *ZEB2* from eQTL data in aortic tissue in our analyses (Supplementary Table S4).

At the second ASI locus, *FOXO1* represents a good candidate gene, rs7331212 is located within the O class of the forkhead family of transcription factors. There is genome-wide significant association of the same variant with pulse wave PPT^21^, which is the time interval between the peak values of the direct and reflected components of the pulse waveform using to calculate ASI^28^. Although the specific function of *FOXO1* gene is not well described, it may play a role in blood pressure regulation. Specifically, lack of *FOXO1* has been shown to reduce expression of angiotensinogen, which is a precursor of angiotensin II that mediates vasoconstriction, in knockout mouse models^29^. Furthermore, *FOXO1* is involved in the signalling axis that regulates mindin^30^, which has a role in neointima formation where there is vascular smooth muscle cell proliferation. Importantly, the significance of the association between our lead variant at *FOXO1* and ASI persisted after adjustment for MAP in our study.

At our third ASI locus, there are few candidate genes. *C1orf21* is an uncharacterised protein-coding gene that at present has not been functionally annotated.

At the 4^th^ locus rs10840457 is located near the *MRVI1* (Murine Retrovirus Integration Site 1 Homolog, also known as IP3R-associated cGMP kinase substrate (*IRAG*)). The *MRVI1* gene is responsible for encoding the *MRVI1*/*IRAG* protein, which is present in a number of tissues including aorta and trachea^31^, and is involved in smooth muscle contractility. Specifically, there is inhibition of calcium release from endoplasmic reticulum following co-expression of *IRAG* and cGMP-dependent protein kinase type Iβ (cGKIβ) in the presence of cGMP^31^. In a study using *IRAG*-knockout mice, the authors concluded that signalling of cGKIβ via *IRAG* is a vital functional component in the regulation of smooth muscle tone and intracellular calcium by nitric oxide and atrial natriuretic peptide^32^. At this locus *MRVI1* represents an interesting candidate, but we note this is a locus that was not genome-wide significant in the secondary analyses, thus further validation will be required.

Gene-based testing by MAGMA revealed *COL4A2* as the most significant gene association with ASI in our cohort, with *FOXO1*, *MRVI1* and *FOXO3* also significant. The lead SNV from the GWAS at the *COL4A2* locus is rs9521719 (*P* = 1.1 × 10^−7^). *COL4A2*, along with the adjacent gene *COL4A1*, encodes the protein subunits of type IV collagen forming heterotrimers. Type IV collagen is a vital structural component of basement membranes and mutations in these genes are seen in disorders such as myopathy, intracerebral haemorrhage and glaucoma^33^. A previous GWAS reported rs3742207 located near the *COL4A1* to have strong association (*P* = 7.08 × 10^−7^) with PWV in a cohort comprising of 4,221 Sardinian individuals^34^. This variant was successfully replicated internally in 1,828 individuals and also in 813 Amish individuals. The PWV variant rs3742207 did not show any association with ASI in our cohort (*P* = 0.95). This result may add additional support on there being a difference in the genetic mechanism of ASI compared to PWV, both markers of arterial stiffness.

We found the genome-wide SNV heritability of ASI to be 6%. This estimate is much lower than the reported heritability for carotid-femoral PWV, which ranged between 0.36–0.40^14,35^. This dissimilarity might be explained by the differences in stiffness measure, covariates included and populations. In addition, due to the methodological inconsistencies mentioned above, it should be noted that the heritability of PWV would differ depending on the site of measurement. Mitchell *et al*.^14^ reported moderate heritability for carotid-femoral PWV (h^2^ = 0.40) in their study of 1,480 participants in the Framingham Study offspring cohort. However, when using the less common approach of measuring PWV between the carotid and brachial artery, the heritability estimate was lower (h^2^ = 0.09). In twin studies, the reported heritability estimates range between 0.38 and 0.53^12,13,36^ where the PWV measurements were either located at the wrist (aorto-radial) or foot (aorto-dorsalis-pedis).

The main strength of our study is the very large sample size that, despite the low estimated heritability for ASI, has enabled the identification of genetic variants not found in previous studies. Mean arterial pressure has been shown to have a strong influence on arterial stiffness^15^ so it was included as a covariate in the fully adjusted model, rather than systolic blood pressure (SBP) and diastolic blood pressure (DBP). Despite using the largest cohort to date in identifying genetic variants for arterial stiffness, the main limitation of our study is that loci discovered therein require formal validation in an independent dataset. However, an external study of similar sample size with ASI measurements is currently lacking. We note if we use a more stringent *P*-value for reporting, *P* ≤ 1 × 10^−8^, then two loci would remain significant (*TEX41* and *FOXO1*). In addition, our study cohort consisted of middle-aged individuals of European-ancestry, so our findings may not be generalised to other age groups and ethnic populations.

In conclusion, we identified four loci significantly associated with ASI, an independent predictor of CVD and mortality. The two most significant loci, *TEX41* and *FOXO1*, have SNV associations that may alter arterial stiffness through blood pressure regulation and vascular smooth muscle differentiation. Our results also suggest an important role of calcium in the regulation of smooth muscle tone contributing to arterial stiffness. Further research will be necessary to validate our discovered loci in a separate cohort and their confirmation can potentially lead to the development of risk models that can be used in clinical practice.

## Methods

### Study population

The UK Biobank is a large population-based prospective study of >500,000 participants aged 40–69 years at baseline recruited between 2006 and 2010 in England, Scotland and Wales. During the initial assessment visit, a broad range of biochemical, clinical and genotype data were collected and participants had a number of physical measurements. The UK Biobank study was approved by the North West Multi-Centre Research Ethics Committee and all enrolled individuals have provided written informed consent for collection, storage, make availability of their data for health-related research. All methods were carried out in accordance with the relevant guidelines and regulations.

### Arterial stiffness index measures

Pulse wave ASI (UK Biobank Field 21021), measured in m/s, was derived using the pulse waveform obtained at the finger (preferably index finger of the non-dependent hand though can be placed on any finger or thumb) with an infra-red sensor (PulseTrace PCA 2^TM^, CareFusion, USA). The shape of the volume waveform in the finger is directly related to the time it takes for the pulse waveforms to travel through the arterial tree in the lower body and to be reflected back to the finger. Measurements were made by clipping the device to a finger and the reading is made over 10–15 seconds. As the participants’ heights were unknown until after the recording of the waveform data, the actual ASI values were calculated (ASI = height/PPT) by UK Biobank outside the assessment centre visit.

Data were taken at the UK Biobank Assessment Centres during the baseline recruitment between 29^th^ April 2009 and 1^st^ October 2010 inclusive (Nmax = 169,822). UK Biobank participants are free to withdraw at any time and so 15 individuals were removed based on the application specific list of anonymised IDs. Individuals with absence of notch position in the pulse waveform (Field 4204) were excluded (n = 25,288) as the notch in the digital volume pulse waveform indicates the reflected component of the pressure transmitted and is therefore required to calculate PPT. Outlier ASI values, defined as three inter-quartile ranges below the first quartile or above the third quartile (n = 81), were also removed from analyses. Finally, participants with missing height, weight or blood pressure measurements were removed from the final analyses (n = 848).

### Covariates

Blood pressure was measured using the Omron 705 IT electronic blood pressure monitor (OMRON Healthcare Europe B.V. Kruisweg 577 2132 NA Hoofddorp). SBP and DBP were derived as the mean of the two recorded automated measurements (UK Biobank Fields 4079, 4080), except for 1,141 individuals who only had one recorded reading. MAP estimation was calculated using the traditional formula: MAP = DBP + 1/3(SBP-DBP). Height (Field 50) was measured using a Seca 202 device (Seca, Birmingham, UK). For individuals who reported use of anti-hypertensive medications through questionnaires in UK biobank (Fields 6177, 6153), their SBP and DBP were adjusting by adding 15 mmHg and 10 mmHg respectively to the mean recorded readings^26,37^. The ID numbers of the arterial stiffness devices used were obtained from Field 4206 in UK Biobank.

### Genotypic data

Central QC and imputation of genotypic data performed by UK Biobank has been previously described^38^. Briefly, genotypic data was obtained through either UK Biobank Axiom or UK BiLEVE Axiom arrays (Affymetrix Research Service Laboratory, Santa Clara, California, USA). The Haplotype Reference Consortium (HRC) and the merged UK10K sequencing +1000 Genomes were used as a reference panels for imputation with preference for the HRC panel where SNPs were present in both panels. This study utilised the refreshed genetic dataset made available by UK Biobank in July 2017^38^.

Genetic QC was performed in 164,835 individuals who underwent assessment of arterial stiffness using finger photoplethysmography and had available genotypic data. This process excluded participants with either high missingness or high heterozygosity defined by UK Biobank in their genotypic data (n = 345), as well as those with mis-match between self-reported and inferred sex from the genotypes (n = 116).

A 4-way *k*-means clustering analysis was performed according to data from the first and second principal components (PC1 and PC2) using the ‘pvclust’ R package (version 2.0–0)^39^ to objectively identify the main ethnic groups (White, Asian, African and Chinese) within UK Biobank. “White” participants were defined for those present in the “White” cluster for both PC1 and PC2 analyses. They also needed to match their self-reported ancestry though “mixed, “other” and “missing” were treated as being broad ethnicity. After restriction to European-ancestry only, 127,121 individuals remained. Lastly, we performed SNV-level QC to exclude 237,751 SNVs from genotyped SNVs in these individuals with the following thresholds: minor allele frequency (MAF) of 1%, Hardy-Weinberg equilibrium (*P*-value of 1 × 10^−6^) and missingness of 0.015 using PLINK 1.9^40^. After exclusions, a final total of 546,505 model SNVs was generated for our GWAS analyses.

### Genome-wide association and heritability analyses

We applied rank-based INT on the residuals from the regression of ASI against the phenotypic covariates (i.e., all except genotyping array and PCs) before performing genetic analyses as the distribution did not approximate a normal distribution (Supplementary Fig. S5). The heritability of ASI explained by additive genetic variation was estimated using a variance components method (BOLT-REML)^51^.

We performed three GWASs using BOLT-LMM (v2.3.2) software^41^ across our 546,505 model SNVs and ~9.9 million imputed SNVs with MAF ≥ 1% and imputation information (INFO) score ≥ 0.3. Our primary ASI GWAS was on the INT residuals including the following covariates: age, age^2^, sex, weight, genotyping array (UK Biobank vs UK BiLEVE), device used to obtain pulse waveform, smoking status (current vs non-current smokers), MAP and first 10 principal components (PCs). As a sensitivity analyses, we also performed a GWAS using untransformed ASI values and same covariates as those included in our primary analysis.

A secondary GWAS where MAP was excluded as a covariate was also performed. All GWASs assumed a linear mixed infinitesimal model method under an additive genetic model implemented in BOLT-LMM. This method accounts for cryptic population structure and allows the inclusion of related individuals permitting greater power compared to principal component analysis^41^.

Genome-wide significance was defined as *P*-value ≤ 5 × 10^−8^ and R (version 3.5.1) statistical Software^42^ was used to generate Manhattan plots and QQ plots. Regional association plots were made for genome-wide significant loci using the LocusZoom web-based platform (locuszoom.org) with the hg19/1000 Genomes (Nov 2014) EUR Build.

Conditional analysis was performed for genome-wide significant locus to detect independent association signals using an approximate conditional and joint multiple-SNP (COJO) analysis implemented in genome-wide complex trait analysis (GCTA) tool^43^. A secondary signal was declared if all three of the following conditions were met:

(i) original *P*-value of newly identified variant was <1 × 10^−6^
(ii) ratio between the lead SNV and secondary association *P*-values on a –log_10_ scale is 1.5 or less, (i.e., –log_10_(*P* lead)/−log_10_(*P* sec) < 1.5)
(iii) ratio between the original association and conditional association *P*-values on a –log_10_ scale is 1.5 or less (i.e., –log_10_(*P*)/−log_10_(*P* cond) < 1.5)

### Functional annotation

Summary statistics from our primary model GWAS analyses were uploaded onto the FUMA (v1.3.4c)^18^ web-based application (http://fuma.ctglab.nl/) in order to perform functional annotations via its SNP2GENE function. The default FUMA settings were used whereby significant SNVs (*P* < 5 × 10^−8^), and those that were in LD (r^2^ ≥ 0.6), with a MAF ≥ 0.01 were selected for further annotation. In addition, the maximum distance between LD blocks to merge into a locus was set at <250 kb and the UKB release 2 European was used as the reference panel population. The functional consequences for these SNVs were then obtained with ANNOVAR^44^. For gene-based analysis, the integrated MAGMA v1.06^19^ was utilised in which SNVs are mapped to a gene according their genomic location before gene-level associations with ASI were tested. Here, a *P*-value with Bonferroni adjustment of 0.05/18697 = 2.67 × 10^−6^ was used to define genome-wide significance. For eQTL mapping, this was implemented by the FUMA platform, GTEx v7 database^45^ (http://gtexportal.org/home/), 53 tissue types was used as the gene expression reference data and the significant threshold was defined as false discovery rate (FDR) < 0.05. Genome-wide significant SNVs and those in LD with r^2^ > 0.8 were examined for previously reported GWAS associations with other traits using PhenoScanner^20^, an online search tool containing over 350 million publicly available association results. The significance threshold chosen for reporting therein was *P*-value < 5 × 10^−8^.

## Supplementary information

Supplementary Information

## Data Availability

The UK Biobank Resource is available, via application, to all bona fide researchers undertaking health-related research that is in the public interest. Summary data are available online: www.ukbiobank.ac.uk/data-showcase. Information on accessing the genetic and phenotype data used in this analysis can be found at www.ukbiobank.ac.uk/using-the-resource/.
