# Supplementary material for: Genome-wide association study identifies loci for arterial stiffness index in 127,121 UK Biobank participants
Source: Sci Rep. 2019 Jun 24;9:9143. doi: 10.1038/s41598-019-45703-0 (PMC6591384; doi:10.1038/s41598-019-45703-0)
Supplement: Supplementary file 1 — Supplementary Information [file 41598_2019_45703_MOESM1_ESM.pdf]

SUPPLEMENTARY INFORMATION

Genome-wide association study identifies loci for arterial stiffness index in 127,121 UK Biobank participants

Kenneth Fung, Julia Ramírez, Helen R. Warren, Nay Aung, Aaron M. Lee, Evan Tzanis, Steffen E. Petersen and Patricia B. Munroe

Supplementary Figures S1-S5

Supplementary Tables S1-S5

Supplementary Figure S1 Participant phenotype selection flow chart

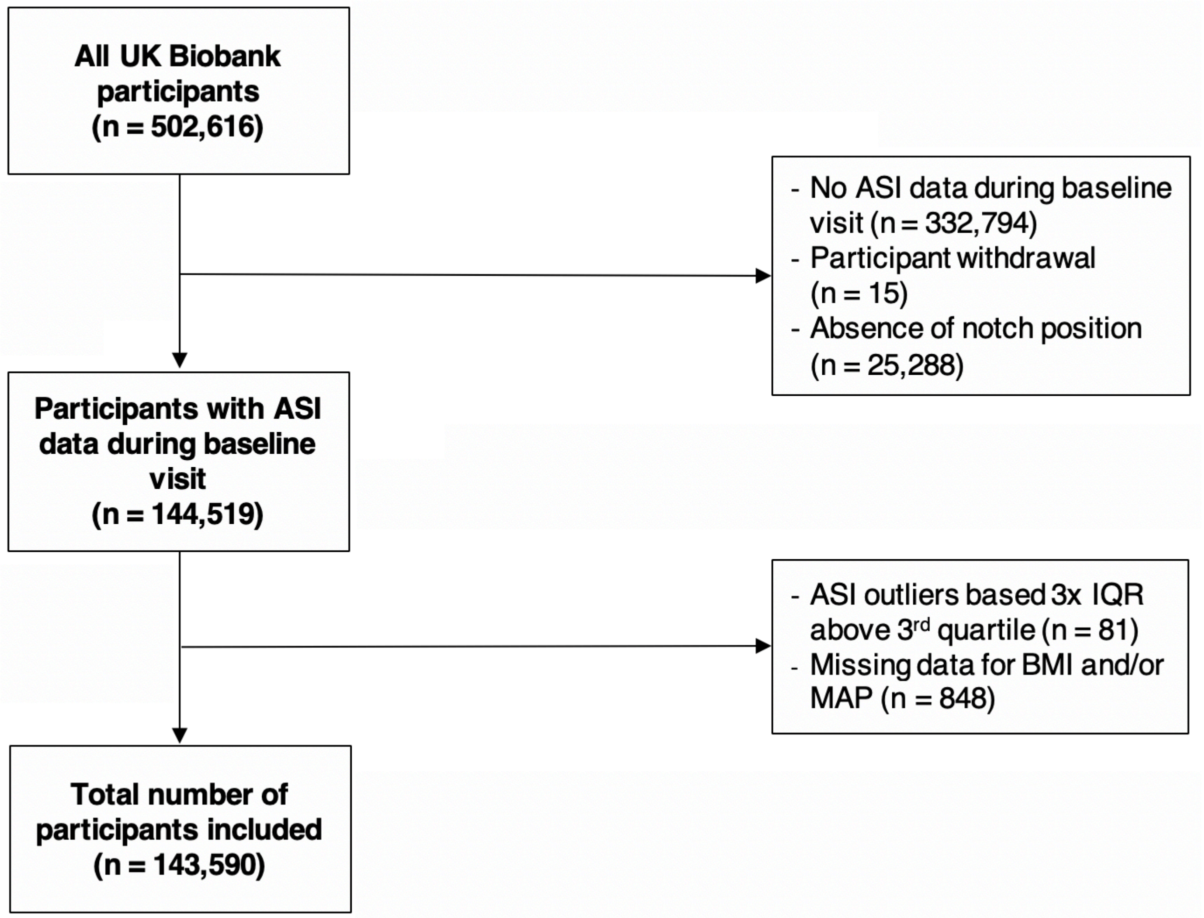

ASI = aortic stiffness index; IQR = interquartile range; MAP = mean arterial pressure; BMI = body mass index.

Supplementary Figure S2 Quantile-quantile (QQ) plots of observed against expected *P*-values for ASI in our primary (left) and secondary (right) models. The genomic inflation factor (λ) value for both is 1.097.

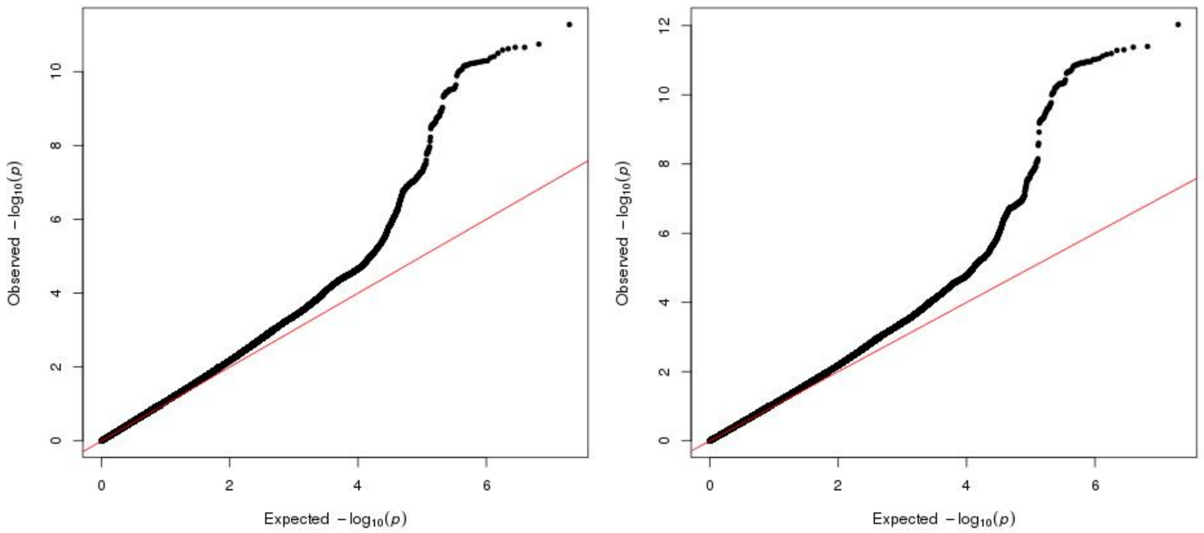

Supplementary Figure S3 Manhattan plot of the results of GWAS with ASI in the secondary model. The red line indicates the *P*-value threshold for genome-wide significance ($P < 5 \times 10^{-8}$) while the blue line indicates *P*-value threshold for suggestive significance ($P < 1 \times 10^{-5}$).

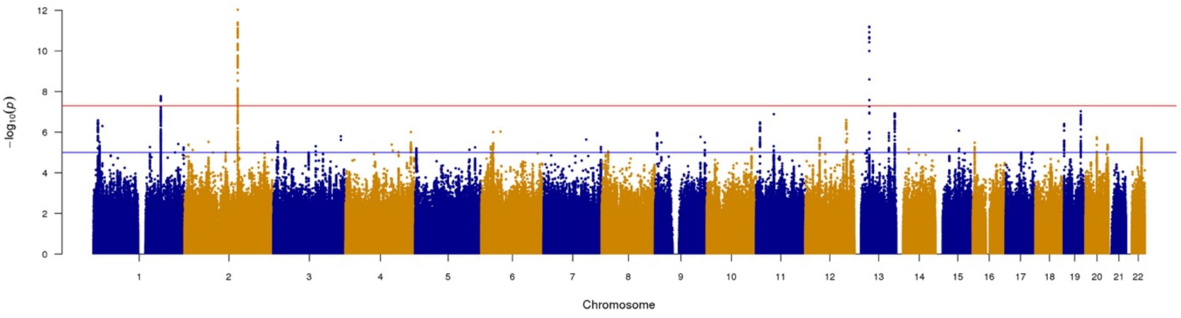

Supplementary Figure S4 Gene-based Manhattan plot of genetic associations with ASI in the primary model. The red dashed line indicates the *P*-value threshold (2.67 x 10^{-6}).

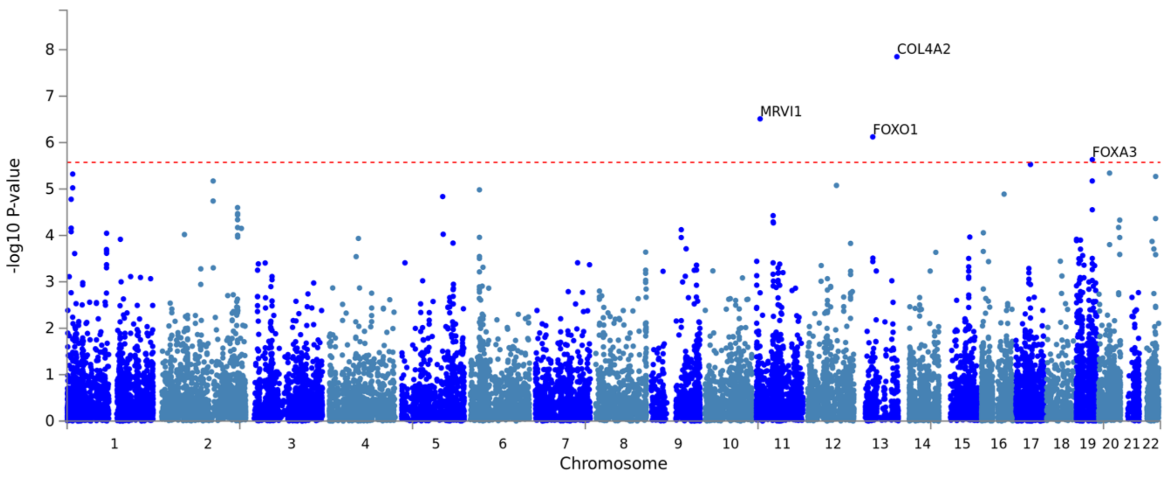

Supplementary Figure S5 Quantile-quantile (QQ) plots of observed ASI against predicted values (left) and observed residuals (from linear regression of ASI) against predicted values (right).

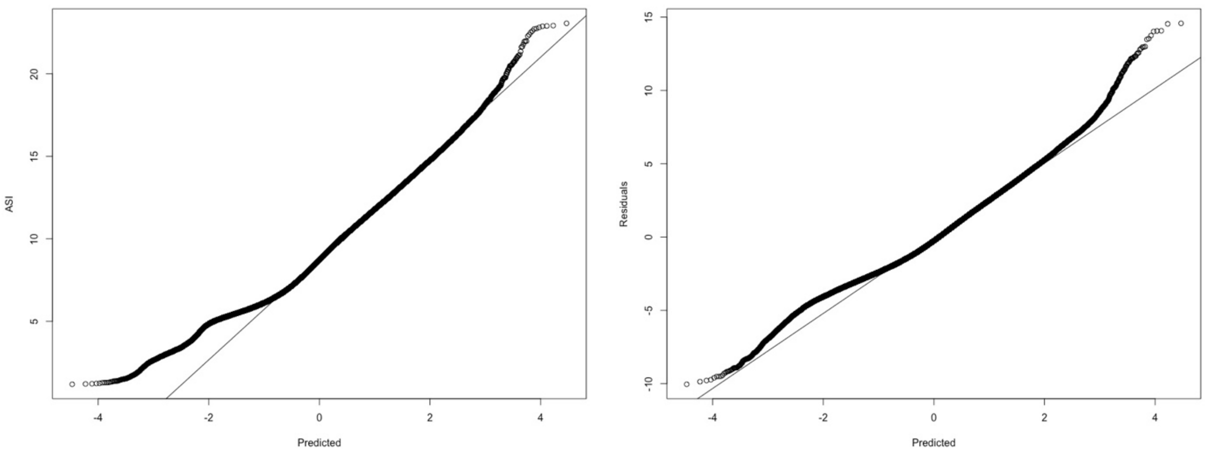

Supplementary Table S1 Baseline characteristics of the 127,121 individuals included in the GWAS analyses. Continuous variables displayed as mean (standard deviation) and categorical data are presented as numbers of participants (percentages)

Parameter	Study group
Age (years)	56 (8.1)
Males	61,199 (48.1)
Height (cm)	170 (9.2)
Weight (kg)	79 (16.0)
UK BiLEVE genetic array	13,326 (10.5)
Systolic blood pressure (mmHg)*	140 (20.3)
Diastolic blood pressure (mmHg)*	84 (11.2)
Mean arterial pressure (mmHg)*	102 (13.4)
Current smokers	8,555 (6.7)
Anti-hypertensive medications	24,264 (19.1)
Arterial stiffness index (m/s)	9.0 (2.7)

*adjusted by adding 15mmHg to systolic blood pressure and 10mmHg to diastolic blood pressure respectively in the presence of anti-hypertensive medications

Supplementary Table S2a Summary of loci associated with arterial stiffness index in the secondary model (excluding mean arterial pressure).

Locus	SNV	CHR	BP	EA	EAF	P	β	SE
C1orf21	rs1930290	1	184272584	T	0.553	1.7E-08	-0.023	0.004
TEX41	rs1006923	2	145775399	T	0.677	9.3E-13	0.030	0.004
FOXO1	rs7331212	13	41185309	G	0.737	6.4E-12	0.031	0.005

Locus is name of the gene in the closest proximity to the most associated SNV.
MAP = mean arterial pressure; SNV = single-nucleotide variant; CHR = chromosome; BP = base pair position (hg19); EA = effect allele; EAF = effect allele frequency; P = *P*-value; β = effect-size estimates on an inverse-normal transformed scale; SE = standard error

Supplementary Table S2b Summary of loci associated with arterial stiffness index using untransformed ASI values

Locus	SNV	CHR	BP	EA	EAF	P	β	SE
C1orf21	rs1930290	1	184272584	T	0.553	5.1E-09	-0.057	0.010
TEX41	rs1006923	2	145775399	T	0.677	1.9E-11	0.070	0.010
FOXO1	rs7331212	13	41185309	G	0.737	2.0E-11	0.074	0.010
RSPH6A	rs8107744	19	46300848	C	0.174	1.4E-08	-0.072	0.013

Locus is name of the gene in the closest proximity to the most associated SNV.
MAP = mean arterial pressure; SNV = single-nucleotide variant; CHR = chromosome; BP = base pair position (hg19); EA = effect allele; EAF = effect allele frequency; P = *P*-value; β = effect-size estimates; SE = standard error

Supplementary Table S3 Loci with suggestive genome-wide significant associations with arterial stiffness index in the primary model ($P < 1 \times 10^{-5}$)

Locus	SNV	CHR	BP	EA	EAF	P	β	SE
CLCN6	rs17037452	1	11895675	A	0.837	9.4E-06	0.024	0.005
HSPB7	rs10927886	1	16339313	C	0.603	6.1E-06	0.018	0.004
FUCA1	rs149320025	1	24167754	G	0.466	4.0E-07	-0.088	0.017
DISC1	rs146306327	1	231909297	A	0.988	5.3E-06	0.094	0.021
NOL10	rs56047999	2	10673316	C	0.876	4.5E-06	0.028	0.006
-	2:65642586_TA_T	2	65642586	-	0.314	3.9E-06	0.020	0.004
ACVR2A	rs151062078	2	148687924	-	0.545	3.6E-06	0.019	0.004
-	2:219518298_CA_C	2	219518298	-	0.429	6.3E-06	-0.018	0.004
BRK1	rs112279447	3	10163420	C	0.907	8.7E-06	0.031	0.007
PPARG	rs17036160	3	12329783	C	0.882	3.9E-06	0.028	0.006
SUSD5	rs7635223	3	33204539	T	0.151	8.4E-06	0.025	0.006
TUSC7	rs13086352	3	116318189	G	0.767	7.2E-06	-0.021	0.005
MAP3K13	rs142358937	3	185145325	C	0.988	1.1E-06	-0.093	0.019
-	4:126534859_GACAA_G	4	126534859	-	0.885	2.4E-06	-0.029	0.006
JADE1	rs17013444	4	129474098	T	0.904	5.6E-06	0.031	0.007
-	4:145463573_CA_C	4	145463573	-	0.824	9.3E-06	-0.024	0.005
-	4:178961198_CCCA_C	4	178961198	-	0.748	8.1E-07	0.023	0.005
C5orf38	rs1494071	5	2947267	G	0.719	9.5E-06	0.020	0.004
LOC102546299	rs149475046	5	163929545	T	0.829	7.5E-06	0.024	0.005
DDR1	rs9501489	6	30783242	G	0.981	4.0E-06	0.067	0.014
MICB	rs9267235	6	31455525	T	0.954	7.3E-06	0.044	0.010
HLA-DRB1	rs2858863	6	32578278	C	0.796	5.3E-06	0.023	0.005
GSTA4	rs11444456	6	52849481	C	0.537	9.3E-07	0.023	0.005
IPCEF1	rs118124932	6	154633543	C	0.964	6.5E-06	0.049	0.011
-	7:116506240_CT_C	7	116506240	-	0.891	2.8E-06	-0.034	0.007
-	8:17485159_ATTTTTAG_A	8	17485159	-	0.922	8.4E-06	0.033	0.008
KIAA1432	rs55850360	9	5605535	G	0.888	7.3E-07	-0.031	0.006
BNC2	rs201551585	9	16900623	C	0.989	4.3E-06	0.091	0.020
DAB2IP	rs7032907	9	124332500	T	0.505	5.0E-06	-0.019	0.004
ABO	rs9411377	9	136145404	C	0.702	9.7E-06	0.020	0.004
OR4A47	rs371147897	11	48531667	-	0.342	1.6E-07	-0.022	0.004
CPNE8	rs7979541	12	39129679	T	0.550	3.1E-06	0.019	0.004
LINC01234	rs41334252	12	114134157	C	0.768	9.3E-07	0.023	0.005
GPC6	rs9524452	13	95003486	G	0.712	1.2E-06	-0.021	0.004
COL4A2	rs9521719	13	111017784	G	0.596	1.1E-07	0.022	0.004
NPAS3	rs2383522	14	34144830	T	0.415	7.6E-06	0.018	0.004
DAPK2	rs55994383	15	64342914	T	0.487	1.4E-06	-0.019	0.004
SEC14L5	rs62036174	16	5015936	G	0.561	6.3E-06	-0.018	0.004
RNF126	rs4919881	19	656701	T	0.155	8.0E-07	0.027	0.005
RSPH6A	rs8107744	19	46300848	C	0.174	1.2E-07	-0.028	0.005
COMMD7	rs780332590	20	31292298	-	0.209	2.7E-06	0.025	0.005
LSM14B	rs6121900	20	60694077	G	0.155	6.7E-06	0.025	0.005

SEPT3	rs62241023	22	42385429	G	0.768	2.7E-06	0.025	0.005

Locus is name of the gene in the closest proximity to the most associated SNV.
MAP = mean arterial pressure; SNV = single-nucleotide variant; CHR = chromosome; BP = base pair position (Build 37); EA = effect allele; EAF = effect allele frequency; P = *P*-value (standard infinitesimal mixed model); β = effect-size estimates on an inverse-normal transformed scale after adjustments for age, age^2, sex, weight, stiffness device used and array, smoking and MAP; SE = standard error

Supplementary Table S4 GTEx analysis of genome-wide significant loci associated with ASI (FDR < 0.05)

Locus	CHR	BP	Risk allele	Tissue	eQTL gene	GTEx P-value
C1orf21	1	184182110	T	Brain – cerebellar hemisphere	APOBEC4	1.87E-05
		184183215	A	Brain – cerebellar hemisphere	APOBEC4	1.88E-05
		184183215	A	Tibial nerve	C1orf21	2.49E-05
TEX41	2	145803003	G	Aorta	ZEB2	3.85E-05
		145811605	A	Aorta	ZEB2	4.23E-05
		145821133	A	Aorta	ZEB2	5.09E-05
		145821717	T	Aorta	ZEB2	4.18E-05
FOXO1	13	41202384	A	Oesophagus – muscularis	SLC25A15	2.18E-05
		41133080	G	Cells – transformed fibroblasts	SLC25A15	2.92E-07
		41143190	A	Cells – transformed fibroblasts	SLC25A15	2.04E-08
		41185309	A	Cells – transformed fibroblasts	SLC25A15	1.36E-06
		41202384	A	Cells – transformed fibroblasts	SLC25A15	5.25E-07
		41209236	G	Cells – transformed fibroblasts	SLC25A15	1.83E-06
		41231556	G	Cells – transformed fibroblasts	SLC25A15	5.08E-06
		41245144	A	Cells – transformed fibroblasts	SLC25A15	3.31E-06
		41185309	A	Skin – sun exposed lower leg	SLC25A15	2.84E-05
		41209236	G	Skin – sun exposed lower leg	SLC25A15	2.40E-05
		41231556	G	Skin – sun exposed lower leg	SLC25A15	8.46E-06
		41245144	A	Skin – sun exposed lower leg	SLC25A15	1.37E-05

Supplementary Table S5 Other trait associations for ASI associated variants using PhenoScanner. Only results from variants in high LD ($r^2 > 0.8$) and *P*-value < 5 x 10^{-8} are shown

SNV	CHR	BP	EA	Trait	Ancestry	N	Beta	SE	P	Study	Reference/ PMID
rs1006923	2	145775399	C	Diastolic blood pressure	European	317,756	-0.016	0.0026	1.42E-09	Neale B	UKBB
rs1006923	2	145775399	C	Mouth or teeth dental problems: dentures	European	336,138	-0.006	0.0010	5.81E-11	Neale B	UKBB
rs1006923	2	145775399	C	Pulse wave arterial stiffness index	European	109,813	-0.027	0.0045	1.36E-09	Neale B	UKBB
rs1006923	2	145775399	C	Pulse wave peak to peak time	European	110,099	0.028	0.0046	1.35E-09	Neale B	UKBB
rs1006923	2	145775399	C	Coronary artery disease	Mixed	547,261	0.035	0.0063	1.71E-08	van der Harst P	29212778
rs10840457	11	10675738	A	Mean platelet volume	European	173,480	0.028	0.0039	9.02E-13	Astle W	27863252
rs10840457	11	10675738	A	Diastolic blood pressure	European	317,756	0.020	0.0027	2.40E-14	Neale B	UKBB
rs10840457	11	10675738	A	Pain type experienced in last month: headache	European	336,650	-0.006	0.0010	2.34E-09	Neale B	UKBB
rs10840457	11	10675738	A	Systolic blood pressure	European	317,754	0.018	0.0026	1.42E-11	Neale B	UKBB
rs7331212	13	41185309	A	Pulse wave peak to peak time	European	110,099	0.028	0.0048	6.12E-09	Neale B	UKBB

SNV = single-nucleotide variant; CHR = chromosome; BP = base position (hg19); EA = effect allele; N = total number of individuals; SE = standard error; P = *P*-value; PMID = PubMed ID; UKBB = http://www.nealelab.is/uk-biobank/

Information on candidate genes highlighted at suggestive ASI loci

In supplementary table S3, interesting candidate genes for ASI at suggestive loci are indicated. Firstly, the *DDR1* locus (rs9501489, $P = 4.0 \times 10^{-6}$) encodes the discoidin domain receptor tyrosine kinase 1. Tyrosine kinase receptors are involved in cell migration, differentiation and extracellular matrix remodelling[1]. Interestingly, *DDR1* receptors have previously been reported to be involved in collagen synthesis. Secondly, the *FUCA1* gene region, where the variant rs149320025 also had suggestive genome-wide significance with ASI ($P = 5.4.0 \times 10^{-7}$). *FUCA1* is a protein encoding gene for alpha-L-fucosidase 1, which is lysosomal enzyme involved in the breakdown of glycoproteins and glycolipids[2]. There are mutations in this gene with associations with lysosomal storage diseases and increased aortic stiffness have been reported in certain groups[3].

Supplementary references

1. Krohn, J. B. *et al.* Discoidin Domain Receptor-1 Regulates Calcific Extracellular Vesicle Release in Vascular Smooth Muscle Cell Fibrocalcific Response via Transforming Growth Factor-β Signaling. *Arterioscler. Thromb. Vasc. Biol.* **36**, 525–33 (2016).
2. Willems, P. J., Seo, H.-C., Coucke, P., Tonlorenzi, R. & O'Brien, J. S. Spectrum of mutations in fucosidosis. *Eur. J. Hum. Genet.* **7**, 60–67 (1999).
3. Wens, S. C. A. *et al.* Increased aortic stiffness and blood pressure in non-classic Pompe disease. *J. Inherit. Metab. Dis.* **37**, 391–7 (2014).
